# Retraction: The ancestral origin of the critically endangered Quadricorna sheep as revealed by genome-wide analysis

**DOI:** 10.1371/journal.pone.0279019

**Published:** 2022-12-07

**Authors:** Gabriele Senczuk, Marika Di Civita, Luigina Rillo, Alessandra Macciocchi, Mariaconsiglia Occidente, Giorgio Saralli, Valentina D’Onofrio, Tiziana Galli, Christian Persichilli, Claudio Di Giovannantonio, Fabio Pilla, Donato Matassino

After this article [1] was published, the authors identified data analysis errors that led to an overestimation of genomic differentiation among breeds. In light of this issue, the article’s results and conclusions are not valid. Therefore, the authors retract this article.

All authors agree with retraction. We are preparing a revised version of the article in which the errors have been addressed.
